# Acknowledgement to Reviewers of *Healthcare* in 2013

**DOI:** 10.3390/healthcare2010123

**Published:** 2014-02-27

**Authors:** Healthcare Editorial Office

**Affiliations:** MDPI AG, Klybeckstrasse 64, CH-4057 Basel, Switzerland

The editors of *Healthcare* would like to express their sincere gratitude to the following reviewers for assessing manuscripts in 2013:

Alteren, Johanne
Apold, Susan
Barker, Caroline
Bergelson, Jeffery M.
Bungay, Hilary
Büscher, Christian
Chin, K.Y.
Chrysanthaki, Theopisti
Dadras, Sam
Fröhlich, Eleonore
Genders, Roel
Gill, James
Golemati, Spyreta
Grootaert, Charlotte
Grossmann, Kenneth F.
Gutzmer, R.
Hajdarevic, Senada
Hedberg, Berith
Hennen, Leonhard
Hinz, Torsten
Hofbauer, G.F.L.
Hofbauer, Günther
Horton, Mark
Idzenga, Tim
Kapsokalyvas, Dimitrios
Khan, M. Omar F.
Kitzinger, Hugo B.
Klode, Joachim
Kretschmer, Lutz
Leslie, Wilma
Lorenzo, Michele
Ludwick, D.A.
Lymberopoulos, Dimitrios K.
Maldonado, José A.
Morio, Florent
Nehal, Kishwer
Newman, Lareen
O’Brien, Paul E.
O’Malley, Ann S.
Patalay, Rakesh
Peters, Christian Daugaard
Pizzo, Salvatore
Polizzotto, Mark
Posch, Christian
Pu, Lee Q.
Rector, A.
Reimer, Holly
Renz, Susan
Shore, Jay
Silva, Edibaldo
Testori, Alessandro
Timmins, Fiona
Tran, Tung T.
Vaccher, Emanuela
Van Besouw, Nicole M.
Van Veldhuizen, Peter J.
Voit, Christiane
Yaroslavsky, Anna N.

